# Complete genome sequence of *Butyricimonas faecihominis* JCM 18676^T^

**DOI:** 10.1128/MRA.00514-23

**Published:** 2023-09-05

**Authors:** Hironori Fukuoka, Dieter M. Tourlousse, Mayu Hamajima, Kazuyoshi Koike, Itaru Endo, Yuji Sekiguchi

**Affiliations:** 1 Department of Gastroenterological Surgery, Yokohama City University, Kanagawa, Japan; 2 Biomedical Research Institute, National Institute of Advanced Industrial Science and Technology (AIST), Tsukuba, Ibaraki, Japan; Wellesley College, Wellesley, Massachusetts, USA

**Keywords:** human gut microbiota, type strain, genome sequence, *Butyricimonas faecihominis*

## Abstract

We report a complete genome sequence of *Butyricimonas faecihominis* JCM 18676^T^, generated by nanopore sequencing. The genome consists of a single circular chromosome of 4,851,806 bp, with a G + C content of 42.9%, and was predicted to contain 15 rRNA and 61 tRNA genes and encode for 3,946 proteins.

## ANNOUNCEMENT

The genus *Butyricimonas* currently contains five validly described species of Gram-negative anaerobic bacteria that are often detected in the gastrointestinal tract of mammals, including humans (1). Members of the genus are known to produce short-chain fatty acids, including butyrate (1), and studies in mice have shown that treatment of metabolic disorders with metformin (2) and statins (3) correlates with increased abundance of *Butyricimonas* spp. In humans, the genus was shown to correlate with triglyceride levels and body mass index (4). Opposite to these beneficial properties, *Butyricimonas* spp. have been associated with colorectal adenoma/carcinoma (5, 6). To enable a better understanding of the role(s) of members of the *Butyricimonas* genus in the human gut, we used nanopore sequencing to generate a complete genome assembly of the type strain of *Butyricimonas faecihominis* (JCM 18676^T^ = CCUG 65562^T^ = DSM 105721^T^ = 180–3^T^), isolated from human feces (1).

Cells were obtained from the Japan Collection of Microorganisms (JCM) as L-dried cultures and cultured directly in peptone yeast extract glucose medium at 37°C under an atmosphere of N_2_/CO_2_ [80/20 (vol/vol)]. Extraction of DNA was performed using the MagAttract HMW DNA Kit (Qiagen). Libraries for sequencing were prepared with ONT’s Native Barcoding Kit 24 (SQK-NBD114.24, with Native Barcode 12), without fragmentation and following the manufacturer’s instructions. Sequencing was performed on an R10.4.1 flow cell using a GridION device (translocation speed: 400 bp per second; sampling frequency: 4,000 per second). Default parameters were used for all bioinformatics analyses unless indicated. Base calling was performed using Dorado v0.2.1 (https://github.com/nanoporetech/dorado) with model dna_r10.4.1_e8.2_400bps_sup@v4.0.0. Chimeric and/or concatenated reads were identified and split using Duplex Tools v0.3.1 (https://github.com/nanoporetech/duplex-tools) for four iterations with option -allow_multiple_splits. Libraries were demultiplexed using Guppy’s guppy_barcoder command (v6.4.6, options -enable_trim_barcodes -barcode_kits SQK-NBD114-24). Reads with an average base quality score of <12 and length of <1,000 bases were discarded using NanoFilt v2.8.0 (7), followed by extraction of a set of high-quality reads (150× estimated coverage) using Filtlong v0.2.0 (https://github.com/rrwick/Filtlong), with option -mean_q_weight 10. This yielded a total of 104,904 reads (727,779,739 bases) with an N50 value of 10,340 bases. Genome assembly was performed using Flye v2.9.1 (8); this yielded a single circular contig, as indicated by Flye, without overlap at the contig ends (as verified by local alignment). Polishing of the assembly was performed with Medaka v1.7.3 (https://github.com/nanoporetech/medaka) using model r1041_e82_400bps_sup_v4.0.0, after rotation of the assembly using SeqKit’s restart command (v2.2.0), (9). Completeness (99.5%) and contamination (0.5%) were confirmed with CheckM v1.1.3 (10) using lineage-specific marker gene sets (lineage_wf). DDBJ’s Fast Annotation and Submission Tool v1.2.18 (11) was used for annotation.

The genome of *B. faecihominis* JCM 18676^T^ was assembled as a single circular chromosome with a length of 4,851,806 bp and a 42.9% G + C content. Annotation predicted 15 ribosomal RNA genes, 61 transfer RNA genes, and 3,946 protein-coding sequences. The availability of a complete genome sequence of *B. faecihominis* JCM 18676^T^ will help us understand the function and role of *B. faecihominis* in the human intestinal tract and may clarify its association with various diseases.

## Data Availability

The genome sequence has been deposited in DDBJ/EMBL/GenBank under the accession number AP028155. Sequencing reads are available in DDBJ’s Sequence Read Archive (DRA) under accession number DRR462685.
